# Structural Basis for Binding of Neutralizing Antibodies to *Clostridioides difficile* Binary Toxin

**DOI:** 10.1128/jb.00456-22

**Published:** 2023-03-23

**Authors:** Jory A. Goldsmith, Vincent Dewar, Philippe Hermand, Normand Blais, Jason S. McLellan

**Affiliations:** a Department of Molecular Biosciences, The University of Texas at Austin, Austin, Texas, USA; b GSK, Rixensart, Belgium; University of Illinois at Chicago

**Keywords:** *Clostridioides difficile*, X-ray crystallography, neutralizing antibodies, pore-forming toxins

## Abstract

Clostridioides difficile is a Gram-positive opportunistic human pathogen that causes 15,000 deaths annually in the United States, prompting a need for vaccine development. In addition to the important toxins TcdA and TcdB, binary toxin (CDT) plays a significant role in the pathogenesis of certain C. difficile ribotypes by catalyzing the ADP-ribosylation of actin in host cells. However, the mechanisms of CDT neutralization by antibodies have not been studied, limiting our understanding of key epitopes for CDT antigen design. Therefore, we isolated neutralizing monoclonal antibodies against CDT and characterized their mechanisms of neutralization structurally and biochemically. Here, 2.5-Å and 2.6-Å resolution X-ray crystal structures of the antibodies BINTOXB/22 and BINTOXB/9, respectively, in complex with CDTb—the CDT subunit that forms a heptameric pore for the delivery of toxic CDTa enzyme into the host cytosol—showed that both antibodies sterically clash with adjacent protomers in the assembled heptamer. Assessment of trypsin-induced oligomerization of the purified CDTb protoxin *in vitro* showed that BINTOXB/22 and BINTOXB/9 prevented the assembly of di-heptamers upon prodomain cleavage. This work suggests that the CDT oligomerization process can be effectively targeted by antibodies, which will aid in the development of C. difficile vaccines and therapeutics.

**IMPORTANCE** Clostridioides difficile strains associated with worse clinical outcomes have been found to secrete a toxin called CDT (or binary toxin). As blocking the function of this toxin could help mitigate C. difficile infections, we sought to determine the molecular basis for the inhibition of CDT by monoclonal antibodies. We isolated monoclonal antibodies targeting the B-component of CDT (CDTb) and selected two with neutralizing activity for detailed structural and biochemical characterization. High-resolution crystal structures of each antibody bound to CDTb showed that their presence would preclude the assembly of a CDTb oligomer required for activity. Oligomerization of CDTb *in vitro* was shown to be blocked in the presence of the neutralizing antibodies, but not a control antibody.

## INTRODUCTION

The sporogenic, anaerobic, Gram-positive enteropathogen Clostridioides difficile is a predominant cause of nosocomial intestinal infections. C. difficile infection (CDI) develops opportunistically when antibiotic treatment damages the host commensal microbiota (1, 2). Symptoms of CDI range from mild diarrhea to more severe or life-threatening complications, such as pseudomembranous colitis, toxic megacolon, and death (3), which originate from neutrophilic inflammation within the colonic mucosa and lumen (4). C. difficile has been associated with substantial morbidity and mortality in all age groups worldwide (5). In the United States, C. difficile is the most common cause of health care-associated infections, accounting for approximately 15% (6). This represented an estimated half a million infections and 29,000 deaths in 2012, with 80% of these deaths occuring in patients over 65 years old (7). In Europe, the burden of health care-associated CDI reaches 124,000 cases annually (8), and this quantity is likely an underestimate (9). Overall, these data highlight the important impact of C. difficile infections.

Two C. difficile exotoxins, namely, TcdA and TcdB, are important virulence factors responsible for the symptoms associated with C. difficile infection (10, 11). However, some of the most virulent clinical strains also produce a third toxin termed the C. difficile transferase toxin (CDT, or binary toxin), a member of the iota toxin family (12, 13). CDT is composed of two separated subunits, CDTa and CDTb, working in conjunction to exhibit the toxic effect (14). CDTb is the cell-binding protein that assembles into pore-forming heptamers. Upon CDTb pore formation within the host endosomal membrane, the catalytically active CDTa cargo is transported into the cytoplasm. Subsequent ADP-ribosylation of G-actin by CDTa results in depolymerization of the actin cytoskeleton and protrusion of host cell membrane segments, which may favor C. difficile adherence (15, 16). Because binary toxin-producing strains are associated with negative clinical outcomes (12), CDT should be explored as a C. difficile vaccine target.

The structure of the CDTb heptamer is known in multiple conformational states, including the prepore state and pore state (17, 18). Mature CDTb consists of five domains: D1, D2, D3, D3′, and D4 (17). D1 prevents premature heptamerization and binds CDTa (18, 19), whereas D4 mediates binding to the host cell receptor (20). D3′ interacts with glycans, and D2 and D3 form the heptamerization interface and β-barrel pore (17). The CDTb protoxin (proCTDb) additionally contains an N-terminal prodomain that is cleaved by cell-surface proteases to yield active CDTb (17, 21). Despite recent structural advances, no monoclonal antibodies against CDT have been studied, and the mechanisms of antibody-mediated inhibition of CDT function are not well understood. The delineation of key neutralizing epitopes and mechanisms of antibody-mediated neutralization is therefore desired because this information can be used to optimize CDTb antigens for the elicitation of neutralizing responses.

To gain insight into the antibody-mediated neutralization of CDT, we isolated and characterized monoclonal antibodies against CDTb. Their neutralization activity was evaluated in a cell cytotoxicity assay and their binding affinity by surface plasmon resonance. Structural analyses, including electron microscopy and X-ray crystallography, further revealed that both antibodies bind distinct oligomerization interfaces of mature CDTb (17, 18). This work provides key insights for CDT vaccine design and lays the foundation for the development of antibody therapeutics targeting CDT.

## RESULTS

### Immunization of mice with proCDTb yields toxin-neutralizing antibodies.

**FIG 1:**
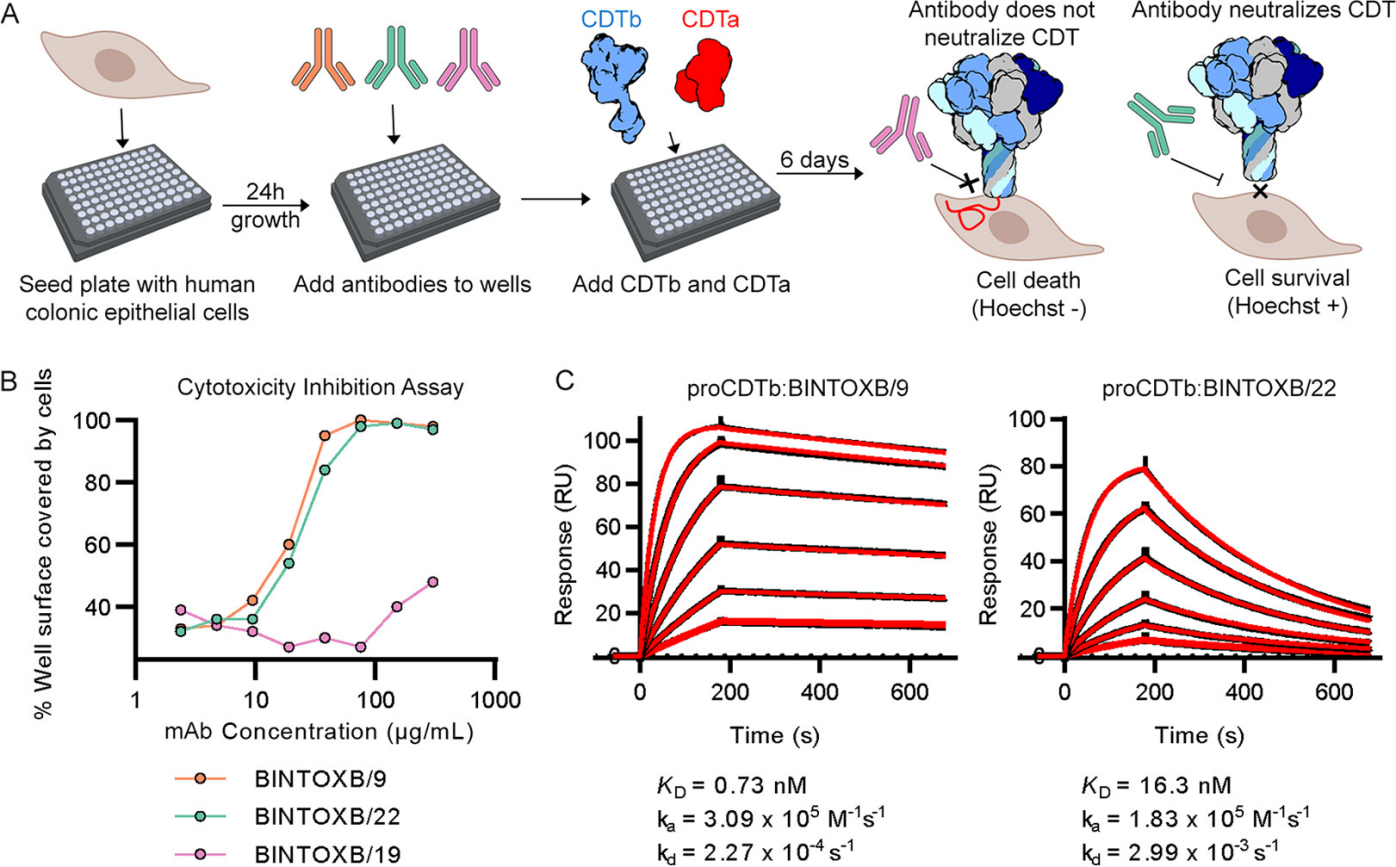
Monoclonal CDTb antibodies neutralize CDT. (A) CDT neutralization assay scheme based on cell survival. (B) Neutralization curves for BINTOXB/9 IgG (orange circles), BINTOXB/22 IgG (green circles), and BINTOXB/19 IgG (pink circles). (C) Surface plasmon resonance sensorgrams for BINTOXB/9 Fab and BINTOXB/22 Fab binding to proCDTb. Black traces show measured response and red traces show the kinetic fit to a 1:1 binding model. The association phase was 180 s and the dissociation phase was 600 s. CDT, C. difficile transferase toxin; *K_D_*, equilibrium dissociation constant; *k_a_*, association rate constant; *k_d_*, dissociation rate constant.

The cleaved, monomeric form of CDTb (CDTb residues 210 to 876) was generated by treating proCDTb (CDTb residues 43 to 876) with α-chymotrypsin and used to immunize BALB/c mice, from which hybridomas were generated and screened for antigen binding. Three positive clones (BINTOXB/9, BINTOXB/22, and BINTOXB/19) were isolated and subcloned for further characterization. To assess the neutralization activity of the isolated monoclonal antibodies, we performed a cell cytotoxicity assay in the presence of IgG (Fig. 1A). In this assay, human colonic epithelial cells were grown in a 96-well plate. Addition of CDT to the cells results in their death, which can be detected by the absence of a Hoechst stain signal (Fig. 1A). Incubation of cells with CDT resulted in only ~40% of well surfaces being covered in Hoechst-positive cells at the lowest IgG concentration for all antibodies. However, wells containing increasing amounts of BINTOXB/9 and BINTOXB/22 exhibited concentration-dependent protection of cells from death, with 50% effective concentration (EC_50_) values of 13 μg/mL and 21 μg/mL, respectively (Fig. 1B). In addition, above the EC_50_ for these three neutralizing antibodies, nearly 100% of the wells remained covered by live cells after CDT incubation. However, the presence of BINTOXB/19 during incubation of cells with CDT did not result in a detectable increase in cell survival at any of the concentrations tested.

To understand the relationship between antibody affinity and the observed neutralization, we used SPR to measure Fab binding kinetics (Fig. 1C). The equilibrium dissociation constants (*K*_D_) for BINTOXB/9 and BINTOXB/22 binding to proCDTb were determined to be 0.73 nM and 16.3 nM, respectively. In contrast, the binding of BINTOXB/19 to proCDTb was very weak, with a dissociation constant of 1.3 μM (Fig. S1A in the supplemental material). Despite the relatively weak affinity, proCDTb could be immunoprecipitated using BINTOXB/19 IgG, and BINTOXB/19 Fab co-eluted with proCDTb by size exclusion chromatography (SEC) after being concentrated to ~60 μM proCDTb and ~120 μM BINTOXB/19 (Fig. S1B). Thus, although BINTOXB/19 does interact with proCDTb, it may be unable to neutralize CDT due to its low affinity.

### D4 of CDTb is flexible and contains the BINTOXB/9 epitope.

**FIG 2:**
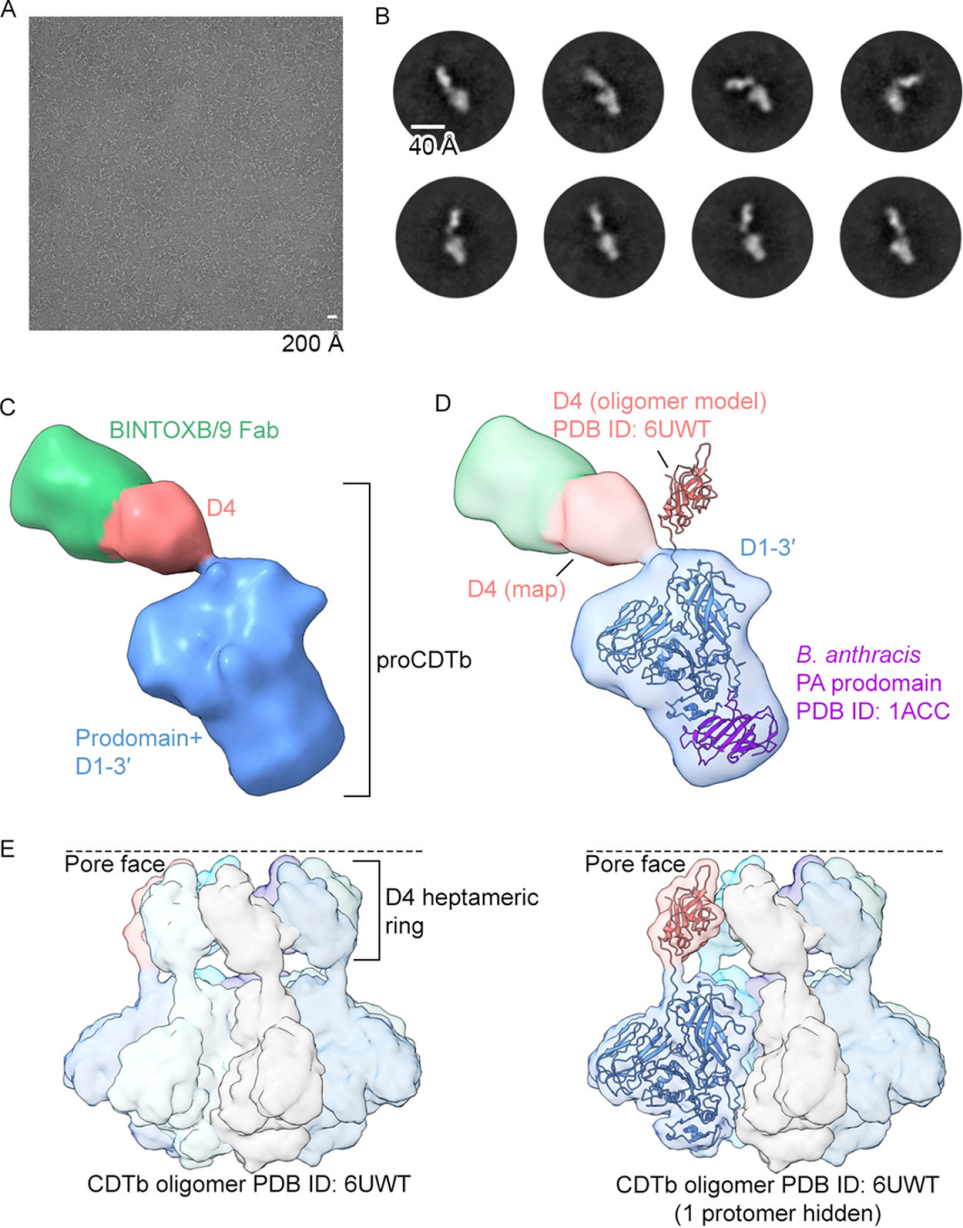
BINTOXB/9 binds to the flexible D4. (A) Negative-stain electron microscopy (nsEM) micrograph of proCDTb+BINTOXB/9 Fab. (B) Representative 2D class averages of proCDTb+BINTOXB/9 particles. (C) nsEM 3D reconstruction of CDTb+BINTOXB/9 Fab. CDTb prodomain and D1-D3′ are colored blue, proCDTb D4 is colored pink, and BINTOXB/9 Fab is colored green. (D) 3D reconstruction from panel C with a transparent surface and fitted models. The mature CDTb model (D1-D4; blue and pink) was obtained from the oligomer structure (PDB ID: 6UWT) and is shown as ribbons. The prodomain model (purple) was obtained by aligning the Bacillus anthracis protective antigen (PA) protoxin structure (PDB ID: 1ACC) to mature CDTb. (E) Cleaved, mature CDTb prepore heptamer (PDB ID: 6UWT) with each protomer shown as a molecular surface. Right: cleaved, mature CDTb prepore heptamer with 1 protomer hidden. One protomer is shown as both ribbon and molecular surface, with D1-D3′ colored blue and D4 colored pink.

To gain insight into the mechanism of neutralization for BINTOXB/9 and BINTOXB/22, we attempted to crystallize proCDTb in complex with BINTOXB/9 Fab or BINTOXB/22 Fab. However, exhaustive screening of crystal conditions suggested that proCDTb-Fab complexes were resistant to crystallization. Therefore, we employed negative-stain electron microscopy (nsEM) for low-resolution epitope mapping of the BINTOXB/9 epitope. 2D classification of particles extracted from nsEM micrographs of the proCDTb-BINTOXB/9 Fab complex revealed that BINTOXB/9 was bound to a small globular domain that was flexibly linked to the rest of proCDTb, with the flexible linker adopting different angles in different 2D class averages (Fig. 2A, B). 3D classification and homogeneous refinement yielded a 3D reconstruction of the complex with one of the linker conformations (Fig. 2C). We generated a proCDTb model by aligning the Bacillus anthracis protective antigen (PA) protoxin (PDB ID: 1ACC) to a cleaved CDTb protomer from an oligomer cryo-EM structure (PDB ID: 6UWT) (18) (Fig. 2D). The proCDTb model exhibited a qualitatively good fit in the nsEM reconstruction, except for proCDTb D4, which is linked to D1-D3′ via a flexible linker (Fig. 2D). The conformation observed for the D3-D4 linker in the nsEM reconstruction of proCDTb differs from the linker conformation adopted between D3 and D4 in the cleaved, mature CDTb protomer within the assembled heptamer, where D4 forms a heptameric ring (Fig. 2E). This suggests that in the monomeric proCDTb, the D3-D4 linker is flexible, likely explaining why proCDTb Fab complexes are resistant to crystallization. In addition, the nsEM reconstruction revealed that the BINTOXB/9 Fab was bound to the flexibly tethered D4. This suggested that the crystallization of BINTOXB/9 with CDTb could be facilitated by using a CDTb fragment containing only D4.

### BINTOXB/9 binds to the CDTb D4 oligomerization interface.

**FIG 3:**
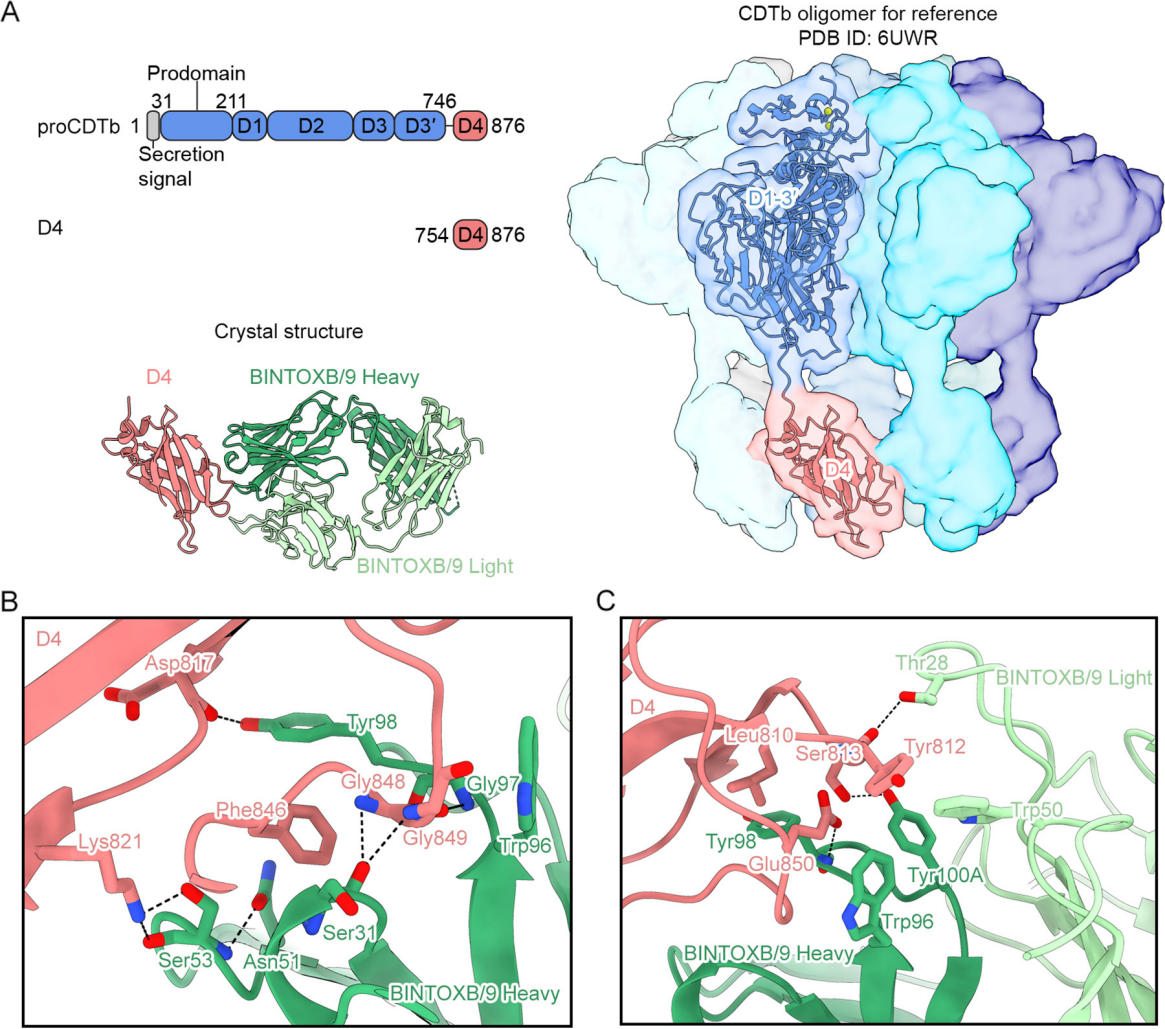
BINTOXB/9 binds to the D4 oligomerization interface. (A) Domain organization of full-length proCDTb and the D4 fragment used for crystallization are shown in the top left, with the secretion signal shown in gray, the CDTb prodomain and D1-D3′ in blue, and D4 in pink. The crystal structure of BINTOXB/9 in complex with the D4 fragment is shown on the bottom left as ribbon representation, with D4 colored pink, BINTOXB/9 Fab heavy chain colored green, and BINTOXB/9 Fab light chain colored pale green. For reference, a model of the CDTb prepore heptamer (PDB ID: 6UWR) is shown on the right, with each protomer shown as a molecular surface. One protomer is shown as both ribbon and molecular surface, with D1-D3′ colored blue and D4 colored pink. (B and C) Zoomed-in views of the BINTOXB/9-D4 interface. BINTOXB/9 heavy chain (green), BINTOXB/9 light chain (pale green) and D4 (pink) are shown as ribbons, with interface residues shown as sticks. Oxygens are colored red and nitrogens are colored blue.

Production of a CDTb C-terminal fragment containing only D4 and complexation of this fragment with BINTOXB/9 Fab yielded crystals in space group *P*2_1_22_1_ which diffracted X-rays to a 2.6-Å resolution. The structure revealed that BINTOXB/9 forms extensive interactions with D4 at the oligomerization interface used to contact D4 of the adjacent protomer in structures of oligomerized CDTb (Fig. 3A). Notably, the complementarity-determining region of the heavy chain (CDRH3) of BINTOXB/9 interacts with Phe846 via Tyr98 (Fig. 3B). Phe846 is a key residue in the D4-D4 interface, where it packs into a hydrophobic pocket formed by Pro790 and Pro835 of the adjacent protomer (Fig. S2). Additionally, a loop in D4 containing Gly848 and Gly849 contacts a hydrophobic patch formed by Tyr828 and Leu829 of the adjacent protomer (Fig. S2). In complex with BINTOXB/9, this loop packs against CDRH3 Trp96, with the Gly848 and Gly849 mainchain atoms, additionally forming hydrogen bonds with the CDRH1 Ser31 mainchain oxygen and CDRH3 Gly97 mainchain nitrogen (Fig. 3B). The BINTOXB/9 epitope also extends to part of the D4 surface that does not directly contact the adjacent protomer. Specifically, CDRH3 Tyr100A, CDRH3 Trp96, and CDRL2 Trp50 form a hydrophobic pocket that interacts with CDTb Tyr812, with the hydroxyl group of Tyr100A also forming a hydrogen bond with the sidechain of CDTb Ser813 (Fig. 3C). CDRL2 Thr28 forms a hydrogen bond with the Ser813 mainchain via its sidechain hydroxyl (Fig. 3C). CDRH3 Tyr98 forms a mainchain hydrogen bond with the CDTb Glu850 carboxyl group and forms a hydrophobic interaction with the CDTb Leu810 sidechain (Fig. 3C). The BINTOXB/9 epitope on CDTb indicates that antibody binding would disrupt the D4-D4 interface.

### BINTOXB/22 interacts with CDTb D3 and D3′.

**FIG 4:**
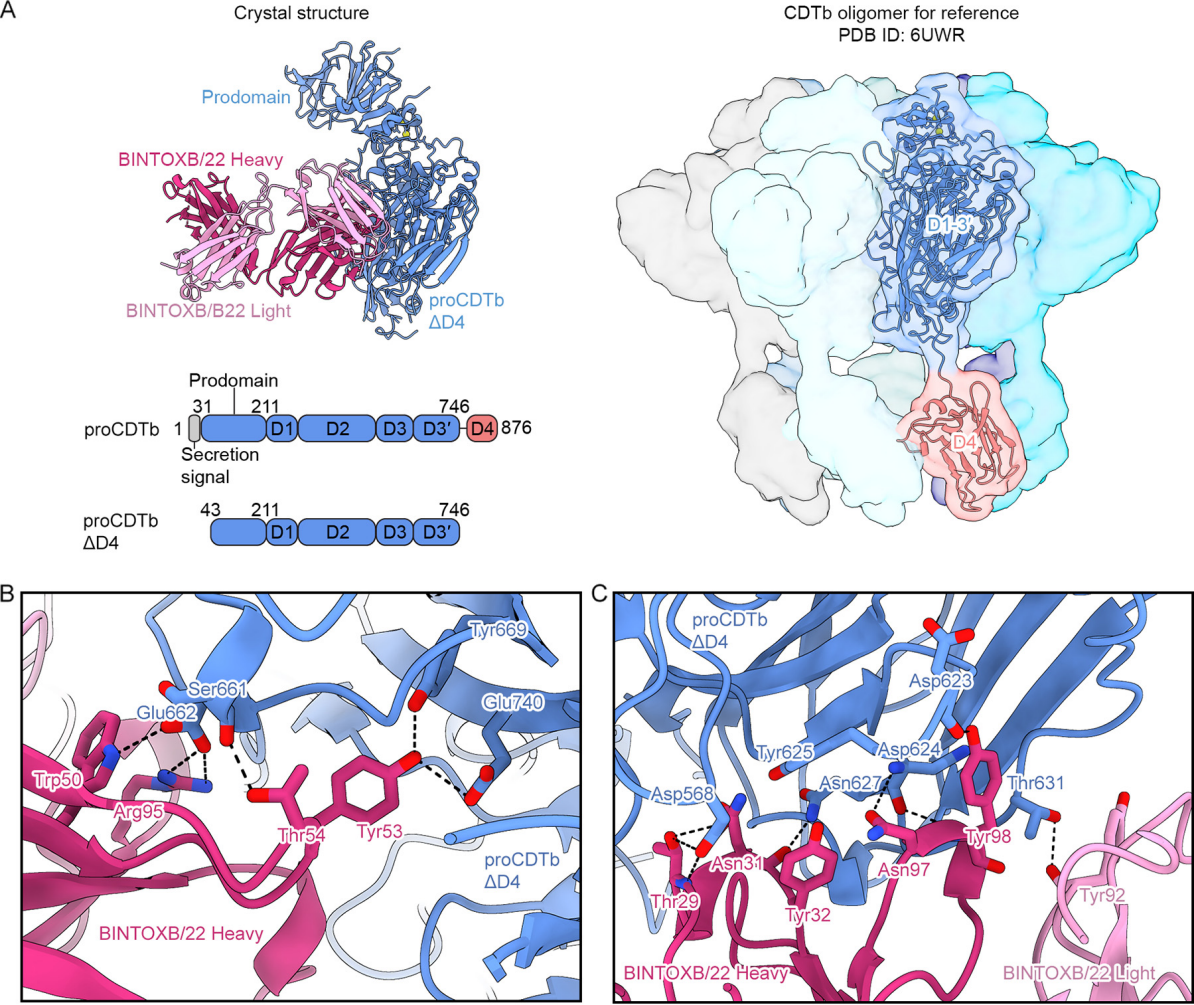
BINTOXB/22 binds to proCDTb D3 and D3′. (A) Crystal structure of BINTOXB/22 in complex with proCDTbΔD4 is shown on the top left as ribbons, with proCDTbΔD4 colored blue, BINTOXB/22 Fab heavy chain colored magenta, and BINTOXB/22 Fab light chain colored pink. Bottom left: domain organization of full-length proCDTb and the proCDTbΔD4 construct used for crystallization, with the secretion signal shown in gray, the CDTb prodomain and D1-D3′ colored blue, and D4 colored pink. For reference, a model of the cleaved, mature CDTb prepore heptamer (PDB ID: 6UWR) is shown on the right, with each protomer shown as a molecular surface. One protomer is shown as both ribbon and molecular surface, with D1-D3′ colored blue and D4 colored pink. (B and C) Zoomed-in views of the BINTOXB/22-proCDTb interface. BINTOXB/22 heavy chain (magenta), BINTOXB/22 light chain (pink), and proCDTbΔD4 (blue) are shown as ribbons, with interface residues shown as sticks. Oxygens are colored red and nitrogens are colored blue.

Unlike BINTOXB/9, BINTOXB/22 does not interact with the D4 fragment by biolayer interferometry (BLI), despite binding to proCDTb (Fig. S3). This suggests that BINTOXB/22 binds to D1-D3′. Therefore, we produced a construct with D4 of CDTb removed, termed proCDTbΔD4 (Fig. 4A), to facilitate crystallization. Consistent with the absence of binding to D4, BINTOXB/22 indeed bound to proCDTbΔD4 as measured by BLI (Fig. S3). Crystals of BINTOXB/22 Fab in complex with proCDTbΔD4 were obtained in space group *P*2_1_2_1_2_1_ which diffracted X-rays to 2.5 Å resolution. After model building and refinement, the structure revealed that BINTOXB/22 binds proCDTb D3 and D3′, a region that does not directly contact adjacent protomers in the context of the cleaved, assembled CDTb oligomer (Fig. 4A). BINTOXB/22 contacts CDTb D3 and D3′ using an interface almost entirely composed of the antibody heavy chain (Fig. 4B, C). Specifically, CDTb Glu662 forms hydrogen bonds between its sidechain carboxyl group and the sidechains of CDRH2 Trp50 and CDRH3 Arg95 of BINTOXB/22 (Fig. 4B). The CDRH2 additionally contacts CDTb by forming a hydrogen bond between Thr54 and the CDTb Ser661 mainchain as well as hydrogen bonds between the Tyr53 sidechain hydroxyl and the Tyr669 and Glu740 sidechains (Fig. 4B). Also, in the CDRH1, Thr29 forms hydrogen bonds with both the sidechain and mainchain of CDTb Asp568, and the mainchain of Asn31 forms a hydrogen bond with the CDTb Asn627 sidechain (Fig. 4C). Lastly, CDRH3 Asn97 forms a hydrogen bond with the mainchain of CDTb Asp624 and the CDRH3 Tyr98 mainchain forms a hydrogen bond with the mainchain of CDTb Asn627 (Fig. 4C). The only contact formed by the BINTOXB/22 light chain with CDTb is a hydrogen bond formed by the mainchain carbonyl oxygen of CDRL3 Tyr92 with the sidechain hydroxyl group of CDTb Thr631 (Fig. 4C). None of the residues contacted by BINTOXB/22 on CDTb are part of the oligomerization interface (17, 18).

**FIG 5:**
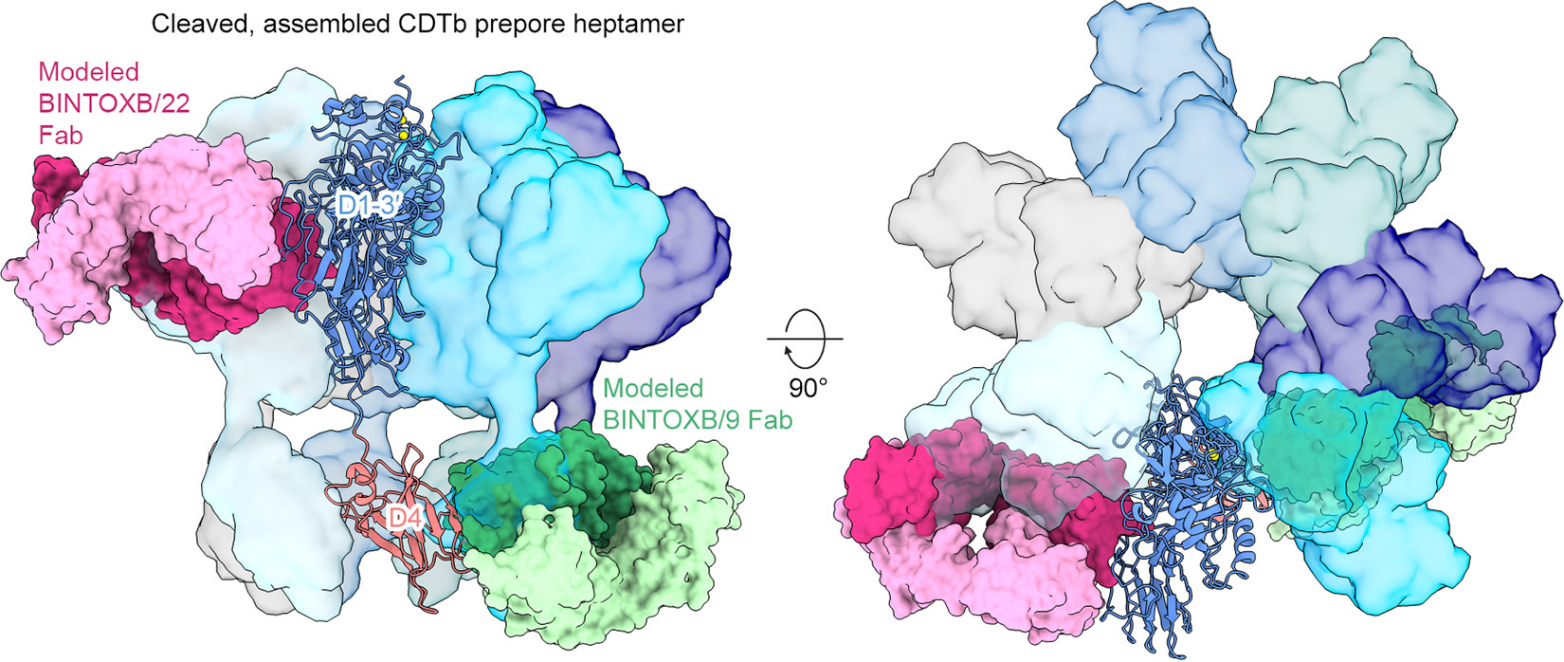
BINTOXB/9 and BINTOXB/22 clash with adjacent protomers in the cleaved, mature CDTb heptamer. The cleaved, mature CDTb prepore heptamer (PDB ID: 6UWT) is shown with 6 protomers shown as molecular surfaces and one protomer as a ribbon. BINTOXB/9 (green) and BINTOXB/22 (purple) are modeled bound to the CDTb protomer, shown as ribbons, based on the Fab-antigen crystal structures.

Although BINTOXB/22 does not directly contact a CDTb oligomerization interface, it is nonetheless expected to prevent CDTb heptamer formation. Modeling of BINTOXB/9 and BINTOXB/22 Fabs into the assembled CDTb heptamer shows how they preclude the formation of distinct homo-oligomerization interfaces (Fig. 5). BINTOXB/22 does not bind the oligomerization interface of D2, but its binding to D3 and D3′ nonetheless results in a steric clash with the adjacent protomer. In contrast, BINTOXB/9 directly contacts the D4-D4 interface and clashes with D4 of the neighboring protomer.

### BINTOXB/22 and BINTOXB/9 inhibit CDTb oligomer formation *in vitro*.

**FIG 6:**
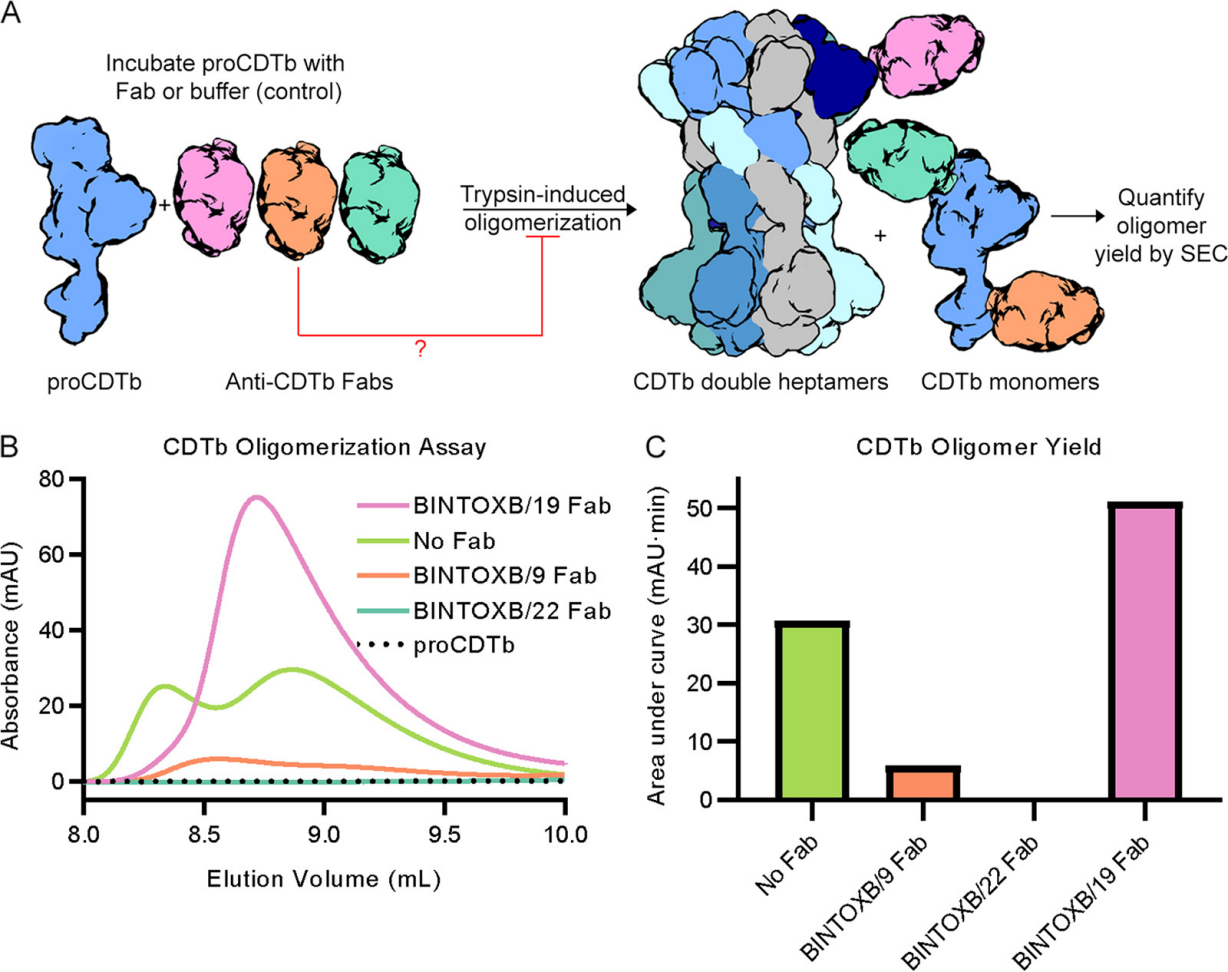
BINTOXB/9 and BINTOXB/22 prevent CDTb oligomerization *in vitro*. (A) Assay for the assessment of CDTb oligomerization *in vitro*. (B) Size exclusion chromatography (SEC) profiles of untreated proCDTb, and proCDTb trypsinized with or without Fabs, showing the elution volume range that contains the expected peak for the cleaved CDTb double heptamer. The trace for the untreated proCDTb is shown as a dotted line. The trace for proCDTb trypsinized with no Fab is shown in green, with BINTOXB/19 Fab in pink, with BINTOXB/9 Fab in orange, and BINTOXB/22 in teal. (C) Quantification of the area under the curve of the oligomer peaks obtained after trypsinization in the presence or absence of Fabs.

The crystal structures of BINTOXB/9 and BINTOXB/22 in complex with CDTb suggest that these antibodies may neutralize CDT by preventing oligomerization of cleaved CDTb into pore-forming heptamers, as has been previously described for a B. anthracis protective antibody (22). To assess the ability of BINTOXB/9, BINTOXB/22, and BINTOXB/19 to prevent assembly, we performed trypsin-induced oligomerization of proCDTb *in vitro* in the presence of antibody. Limited trypsinization of proCDTb in solution has been shown to result in the formation of CDTb double heptamers, which exist as a mixture of symmetric and asymmetric dimers of the pore-forming heptamer (17, 18). We performed trypsin-induced oligomerization in the absence of antibody, as well as in the presence of a 2-fold molar excess of either BINTOXB/9, BINTOXB/22, or BINTOXB/19 Fabs. Formation of the CDTb double heptamer was detected by the appearance of a high-molecular-weight peak in the SEC profile of the trypsinization reaction. As expected, trypsinization of proCDTb yielded a peak for the CDTb double heptamer, compared to the non-trypsinized proCDTb that contained no oligomer (Fig. 6A). Consistent with previous data, only ~5% of the cleaved CDTb oligomerized, with most of the cleaved CDTb eluting by SEC at the expected molecular weight of a monomer (Fig. S4). Notably, the oligomer peak for trypsinized proCDTb also had a higher-molecular-weight shoulder eluting at the approximate SEC void volume (Fig. 6B), which may be aggregated protein. Critically, trypsinization in the presence of BINTOXB/9 Fab almost entirely prevented formation of the CDTb oligomer, and trypsinization in the presence of BINTOXB/22 Fab completely prevented oligomerization (Fig. 6B, C). This shows that, as suggested by their epitopes in the context of the CDTb heptamer, BINTOXB/9 and BINTOXB/22 likely neutralize CDT by preventing heptamer formation. In the presence of BINTOXB/19, a peak for the CDTb double heptamer was detected after trypsinization (Fig. 6A, B). Interestingly, this peak was larger than the oligomer peak obtained after trypsinization with no Fab but did not contain the shoulder at the void volume (Fig. 6B, Fig. S4). This suggests that BINTOXB/19 improved the oligomer yield in this *in vitro* assay. This may be because the binding of BINTOXB/19 affects the access of trypsin to the cleavage site, slowing cleavage kinetics or preventing over-trypsinization and aggregate formation. BINTOXB/19 Fab also did not co-elute with the assembled oligomer (Fig. S4B), possibly due to its low affinity. The CDTb oligomer peaks obtained via trypsinization in the absence of Fab or the presence of BINTOXB/19 Fab were confirmed via nsEM to form the mature double heptamers (Fig. S5) (17, 18). The double heptamer is apparent in the 2D class averages of extracted particles (Fig. S5A), and a 3D reconstruction of the oligomer was obtained for the asymmetric double heptamer that contains one prepore heptamer and one pore heptamer (Fig. S5B) (17). These data demonstrate that the assembly of CDTb double heptamers can be inhibited by BINTOXB/22 and BINTOXB/9 Fabs.

## DISCUSSION

To date, there are no structural or biochemical data available regarding the neutralization mechanisms of anti-CDT antibodies. The oligomerization interface disrupted by BINTOXB/22 binding is conserved among the AB toxin family (12) and is present in cryo-EM structures of the prepore and pore heptamers of B. anthracis PA (23, 24). Therefore, blockade of the physiological CDTb oligomerization interface by BINTOXB/22 likely underlies its inhibition of trypsin-induced oligomerization. This mechanism suggests that the oligomerization process can be effectively targeted, and that this mechanism may be a key component of the protection afforded by CDT immunization. For the well-studied B. anthracis PA, biochemical studies have shown that a variety of neutralization mechanisms exist (25). Notably, multiple neutralizing antibodies have been shown to prevent PA heptamer formation *in vitro* or bind epitopes which suggest that they function via this mechanism (26–28). Although PA is the most well-studied AB toxin, C. perfringens iota toxin is significantly more similar to CDT (80% sequence identity with iota toxin and 36% similarity with PA). Interestingly, a group of competing iota-toxin-neutralizing antibodies was found to bind an epitope that significantly overlaps with the BINTOXB/22 epitope in CDT (29), suggesting that they may also inhibit oligomerization.

Furthermore, BINTOXB/9 binding to CDTb, which would prevent D4 homo-oligomerization, blocked trypsin-induced oligomerization almost as strongly as BINTOXB/22. Notably, D4 is not required for physiological heptamer formation, as proCDTb that lacks D4 can still oligomerize (18) and B. anthracis PA lacks D4 altogether. Therefore, blocking D4 homo-oligomerization would not necessarily be expected to affect heptamer formation. However, BINTOXB/9 binding to CDTb could still prevent heptamer formation if the steric clash with adjacent D4 prevents the conserved interface at D2/D3 from being close enough to adjacent protomers to oligomerize. Alternatively, BINTOXB/9 binding to D4 and prevention of D4 heptameric ring formation could interfere with the dimerization of heptamers into di-heptamers, which likely only occurs upon *in vitro* protease treatment (17, 18). Because single heptamers would elute along with di-heptamers in the oligomer peak, single heptamers were similarly absent in the SEC profile after trypsinization in the presence of BINTOXB/9 (Fig. S4A). However, single heptameric pores have only been observed by cryo-EM when prodomain cleavage was carried out in the presence of detergent, as the transmembrane region of the pore is otherwise only stabilized within the di-heptamer through hydrophobic interactions with the inner face of the D4 heptameric ring of the opposing heptamer (30). Therefore, prevention of D4 heptameric ring formation may simply disrupt the stabilization of the transmembrane regions of extending pores, and without detergent, those single heptamers could aggregate and evade detection by SEC. In the latter case, the neutralizing activity of BINTOXB/9 would be related to a mechanistic step other than heptamerization. D4 is the receptor-binding domain that interacts with the CDT receptor, lipolysis-stimulated lipoprotein receptor (LSR) (20), and the D4 heptameric ring may be required to trigger pore formation upon receptor binding. The role of the D4 heptameric ring in pore formation has been hypothesized based on a large translation of the ring from the prepore and pore state (17). Alternatively, the structure of the D4 heptameric ring may be optimized for avid binding to LSR clusters because toxin binding induces LSR clustering and migration to lipid rafts (31). Therefore, BINTOXB/9 might inhibit pore formation or receptor binding. In addition, though BINTOXB/9 does not interact with the putative receptor-binding surface (17), it is possible that it clashes with LSR indirectly. Although the BINTOXB/9 mechanism is not yet clear, it is notable that D4 is susceptible to neutralization by antibodies. Clarification of the BINTOXB/9 mechanism could yield insight into the function of D4 and the D4 heptameric ring in the CDTb cell intoxication mechanism.

The neutralization exhibited by both BINTOXB/22 and BINTOXB/9 suggests that immunization with the monomeric protoxin may be preferred to immunization with an oligomeric form. Although the oligomer has potential benefits as an immunogen, such as potentially presenting an avid surface with multiple copies of the same epitope or quaternary epitopes, it does not contain the BINTOXB/22 or BINTOXB/9 epitopes. Due to the specificity of BINTOXB/22 and BINTOXB/9 for the CDTb monomer, a CDTb antigen design that precludes oligomerization could favor the elicitation of similar neutralizing antibodies. On the other hand, some neutralizing PA antibodies, such as 1G3 and hmPA6, were raised against the PA heptamer and do not interact with the monomeric protoxin (27, 32). Therefore, in the case of CDTb, further studies are required to assess the distribution of neutralizing epitopes across pre- and post-oligomerization states as well as their relative protection as immunogens. However, targeting the monomeric form may be more effective because the toxin can be bound immediately upon secretion, whereas the heptamer can only be targeted while it is transiently present as an oligomer on the surface of the host cell.

In addition to vaccination, therapeutic antibody treatment has shown promise for C. difficile infection (33–35). BINTOXB/22 and BINTOXB/9 are the first neutralizing monoclonal antibodies described against CDT and they bind to distinct neutralizing epitopes. Development of human monoclonal antibodies that compete with BINTOXB/22 and BINTOXB/9 could therefore allow for the addition of CDT antibodies to therapeutic cocktails containing TcdA and TcdB antibodies (35). Notably, multiple neutralization mechanisms have been observed for PA antibodies, such as prevention of receptor binding, inhibition of prodomain cleavage, prevention of oligomerization, and inhibition of enzyme loading (25). Further research is required to discover additional modes of CDT inhibition by antibodies with diverse mechanisms. Overall, this work defined two distinct neutralization-sensitive epitopes that will facilitate the design of vaccines and therapeutics.

## MATERIALS AND METHODS

### Mouse immunization and hybridoma generation.

Five female BALB/c mice aged 6 to 8 weeks were immunized using the Repetitive IMmunization Multiple Sites (RIMMS) protocol (36). These received 100-μL injections of cleaved, monomeric CDTb antigen at the following doses, 20, 5, 5, and 2.5 μg, subcutaneously at multiple points on days 0, 4, 8, and 11 with GSK adjuvant system 02 (AS02). On day 13, lymph nodes were collected from the 5 mice. After grinding, cells were chemically fused with SP2/0-Ag14 myeloma at a 5:1 ratio using polyethylene glycol 1500 (PEG-1500; Roche) and plated in 96-well cell culture plates at a concentration of 50,000 cells/well. Cells were then cultivated in serum-containing medium at 37°C in a 5% CO_2_ atmosphere. To generate IgG of clones selected for characterization, cell culture supernatants were dialyzed into phosphate-buffered saline (PBS) and sterile-filtered through a 0.22-μm membrane. Antibody content in cell culture supernatant was determined by analytical protein A affinity chromatography using a Hi-Trap Protein A High Performance column (Cytiva 17-0403-03). IgG concentration was determined via absorbance at 280 nm, which was used to calculate the concentration of IgG in the dialyzed supernatant. Dialyzed supernatant containing IgG was then stored at −80°C.

### Clone selection by enzyme-linked immunosorbent assay.

CDTb was directly coated onto Nunc Maxisorp 96-well microplates by incubating 100 μL of 1 μg/mL CDTb in PBS on the plates overnight at 4°C. The coating solution was removed by washing the wells 3 times with 150 mM NaCl, 0.05% Tween 20. Next, 200 μL of blocking solution (PBS, 1% bovine serum albumin [BSA]) was added to the microplate and incubated at room temperature for 30 min. After 3 washes, 100 μL of hybridoma cell culture supernatant was added to the wells and incubated for 30 min at room temperature. After 3 washes, a solution containing biotinylated rabbit anti-mouse IgG (Dako E0413) was added to the wells. After 3 washes, streptavidin-conjugated peroxidase and the colorimetric substrates ortho-phenylene di-amine and hydrogen peroxide (Merck 1.07210.0250) were added to the wells for signal generation. Optical density at 450 nm was determined using a Spectramax 190 Microplate Reader (Molecular Devices).

### Protein expression and purification.

**CDTa and CDTb production for CDT neutralization assay and mouse immunization.** To produce cleaved, monomeric CDTb for mouse immunization and the CDT neutralization assay, the gene encoding proCTDb (CDTb residues 43 to 876) was cloned into pGEX-6p1 (GE Healthcare) downstream of an N-terminal GST (glutathione *S*-transferase) tag and HRV 3C protease cleavage site, and upstream of a C-terminal 6×His tag. To produce CDTa for the CDT neutralization assay, a gene encoding a methionine followed by residues 44 to 463 was cloned into pET24b expression vector (Novagen) upstream of a C-terminal 6×His tag. The constructs were individually transformed into Escherichia coli strain BLR(DE3) pLysS for CDTa construction and BL21(DE3) for CDTb for isopropyl β-d-1-thiogalactopyranoside (IPTG; EMD Chemicals Inc., cat no. 5815)-inducible expression.

To express CDTa and proCDTb, transformants from E. coli strains BLR(DE3) pLysS and BL21(DE3) were inoculated in 200 mL of LB broth, 1% (wt/vol) glucose with 50 μg/mL kanamycin (CDTa in pET24b) or 100 μg/mL ampicillin (proCDTb in pGEX-6p1) and grown overnight at 37°C. The overnight cultures were diluted 1:20 in 500 mL LB medium containing kanamycin (50 μg/mL) or ampicillin (100 μg/mL) and grown at 37°C until the absorbance at 600 nm was equal to ~0.6. The cultures were cooled down before inducing expression with 1 mM IPTG and culture growth for 16 h at 23°C. Cells were then centrifuged at 14,000 rpm for 15 min and frozen at −20°C. The bacterial pellets were resuspended in 50 mM bicine (pH 7.5 or 8.0), 500 mM NaCl, 5 mM Thermo Scientific Pierce TCEP [tris(2-carboxyethyl) phosphine hydrochloride] and a mixture of protease inhibitors (cOmplete, EDTA-free; Roche). Bacteria were lysed by 3 French press passages at 20,000 lb/in^2^. Cell lysates were then clarified by centrifugation at 20,000 × *g* for 30 min at 4°C. The soluble fractions were then loaded on a 5-mL GE HisTrap column (GE) pre-equilibrated with resuspension buffer containing 1 mM TCEP. Resin was washed with 50 mM bicine (pH 7.5), 500 mM NaCl, 10 mM imidazole, and 1 mM TCEP (CDTa) or 50 mM bicine (pH 8.0), 150 mM NaCl, 25 mM imidazole, and 1 mM TCEP (proCDTb). Proteins were eluted using 50 mM bicine (pH 7.5), 500 mM NaCl, 1 mM TCEP, and 500 mM imidazole (CDTa) or 50 mM bicine (pH 8), 150 mM NaCl, 1 mM TCEP, and 250 mM imidazole (proCDTb).

CDTa was further purified by SEC (Superdex 75 Increase column) in 50 mM bicine (pH 7.5 or 8), 150 mM NaCl, and 1 mM TCEP after desalting (Bio-Rad Bio-Gel P-6 Desalting Gel) and concentration (Amicon Ultra centrifugal filter 10 kDa) steps. Fractions were selected based on purity by SDS-PAGE. CDTa protein concentration was determined using a Lowry RC/DC Protein Assay from Bio-Rad. The purified bulk was sterile-filtered at 0.22 μm and stored at −80°C.

To proteolytically cleave GST tag from proCDTb, the sample was desalted (Bio-Rad Bio-Gel P-6 Desalting Gel) into 50 mM bicine, 150 mM NaCl, and 1 mM TCEP. The purified protein was then treated overnight at 4°C with PreScission protease (GE-Healthcare). After treatment, Tween 20 was added to the mixture to achieve a final concentration of 0.2%. To purify the GST-free proCDTb, the treated sample was passed through a GST affinity column (GE GSTrap FF) pre-equilibrated with 50 mM bicine buffer (pH 8.0) containing 150 mM NaCl, 1 mM TCEP, and 0.2% Tween 20. The GST-free protein was then collected from the flowthrough and loaded again on a 5-mL GE HisTrap column (GE) pre-equilibrated with 50 mM bicine (pH 8.0), 150 mM NaCl, 1 mM TCEP, and 0.2% Tween 20. After loading, the column was washed with 50 mM bicine (pH 8), 150 mM NaCl, 0.2% Tween 20, 1 mM TCEP, and 10 mM imidazole. Elution was performed using 50 mM bicine (pH 8.0), 150 mM NaCl, 0.2% Tween 20, 1 mM TCEP, and 500 mM imidazole. After the protein was desalted (BIO-RAD Bio-Gel P-6 Desalting Gel) into 50 mM bicine (pH 8.0), 150 mM NaCl, 1 mM TCEP, and 0.2% Tween 20, cleavage of the proCDTb prodomain was performed using 10% (wt/wt) α-chymotrypsin (bovine pancreas, Sigma-Aldrich). Next, proCTDb and α-chymotrypsin were mixed, and the reaction mixture was incubated for 50 min at room temperature followed by the addition of 2-fold (wt/wt) excess trypsin inhibitor (egg white, Sigma-Aldrich) with respect to the amount of α-chymotrypsin. Cleavage of proCDTb was monitored by SDS-PAGE. Cleaved CDTb protein was further purified by SEC (Superdex 75 Increase column) using 50 mM bicine, (pH 8.0), 300 mM NaCl, and 1 mM TCEP. Fractions containing cleaved, monomeric CDTb were selected based on purity as determined by SDS-PAGE. Protein concentration was determined using a Lowry RC/DC Protein Assay (Bio-Rad). The purified bulk was sterile-filtered at 0.22 μm and stored at −80°C.

**CDTb constructs for biophysical and structural studies.** To generate proCTDb, a gene encoding a methionine followed by CDTb residues 43 to 876 was cloned into pET24b upstream of a 6×His tag. To generate CDTbΔD4, a gene encoding a methionine followed by CDTb residues 43 to 746 was cloned into pET22b upstream of a 6×His tag. To generate D4, a gene encoding a methionine followed by CDTb residues 753 to 876 was cloned into pET22b upstream of a 6×His tag. These 3 constructs were individually transformed into E. coli BL21(DE3) for IPTG-inducible expression. To express protein, 50 mL LB (with ampicillin for proCDTbΔD4 and D4 in pET22b; kanamycin for proCDTb in pET24b) was inoculated with BL21(DE3) transformant and grown overnight at 37°C. The overnight culture was then used to inoculate 1 L of LB-containing antibiotic, and the culture was grown at 37°C until the absorbance at 600 nm was equal to ~0.6. Upon reaching this density, 1 mM IPTG was added, and the culture was grown for 16 h at 16°C. After 16 h, cells were centrifuged for 15 min at 5,000 × *g*. The pellet was then resuspended in 50 mM Tris (pH 8), 200 mM NaCl, 6 mM CaCl_2_, 10 mM imidazole. Cells were then lysed by passage through a Microfluidics LM10 microfluidizer twice at 18,000 lb/in^2^. Lysate was then clarified via centrifugation at 30,000 × *g* for 1 h at 4°C. The soluble fraction was then passed over equilibrated Ni-NTA (nitrilotriacetic acid) resin. The resin was washed with 50 mM Tris (pH 8), 200 mM NaCl, 6 mM CaCl_2_, 20 mM imidazole and protein was eluted using 50 mM Tris (pH 8), 200 mM NaCl, 6 mM CaCl_2_, 150 mM imidazole. proCDTb D4 was further purified using SEC by running it over a Superdex 200 Increase 10/300 GL column (Cytiva) in 2 mM Tris (pH 8), 200 mM NaCl, and 0.02% NaN_3_. proCDTb and proCDTbΔD4 were subjected to ion-exchange chromatography to separate degraded protein from intact protein. For each, the Ni-NTA elution was passed over a HiPrep 26/10 desalting column (Cytiva) and buffer-exchanged into 20 mM bis-Tris (pH 6.5), 6 mM CaCl_2_. Protein was then bound to a HiTrap Q HP anion exchange column (Cytiva) using 20 mM bis-Tris (pH 6.5), 6 mM CaCl_2_ as the running buffer. The column was further washed with running buffer, and elution was achieved using a gradient from 20 mM bis-Tris pH 6.5, 6 mM CaCl_2_ to 20 mM bis-Tris pH 6.5, 6 mM CaCl_2_, 400 mM NaCl over 40 column volumes. Salt gradient elution of proCDTb or proCDTbΔD4 generated two distinct peaks: one containing degradation products eluting at lower salt concentrations, the other containing the intact protein eluting at higher salt concentrations. The intact protein (2nd peak) was collected and run over a Superdex 200 Increase 10/300 GL (Cytiva) SEC column in 20 mM Tris (pH 8), 100 mM NaCl, 6 mM CaCl_2_.

**Antibodies for biophysical and structural studies.** To generate BINTOXB/9, BINTOXB/19, and BINTOXB/22 antibodies, the V_L_ and V_H_ genes obtained from hybridoma sequencing were cloned into pVRC8400 mouse light chain and mouse heavy chain vectors, respectively. Nucleotides encoding an artificial signal peptide (MRPTWAWWLFLVLLLALWAPARG) (37) were added to the N termini of mature V_L_ or V_H_ sequences. The V_L_ was cloned upstream of a mouse kappa light chain C_L_ domain. The V_H_ was cloned upstream of mouse heavy chain C_H_1 and human heavy chain C_H_2 and C_H_3. Heavy chain vectors contain an engineered HRV 3C protease recognition site (LEVFLQGP) in the hinge between C_H_1 and C_H_2, which allows for Fab generation using HRV 3C protease. For the BINTOXB/22 light chain used for crystallography, residues HKTSTSP in the C_L_ were replaced with QGTTS (BINTOXB/22 ‘LCmod’) to favor crystallization (38). To express IgG, heavy and light chain vectors were transiently co-transfected into FreeStyle 293-F cells (Invitrogen). After 6 days, the medium was harvested and run through a 0.22-μm filter. IgG was then purified from the medium by passage over protein A agarose (Thermo Fisher Scientific). To generate Fabs, 1:20 (wt/wt) HRV 3C protease was added to IgG, which was incubated for 1 h at room temperature. Following cleavage, F_C_ was removed by flowing the reaction over protein A agarose (Thermo Fisher Scientific). Fab was then further purified by SEC using a Superdex 200 Increase 10/300 GL column (Cytiva) in 20 mM Tris (pH 8), 100 mM NaCl, 6 mM CaCl_2_.

To obtain complexes of CDTb fragments with Fabs for crystallography, we performed immunoprecipitation using IgG. Either D4 or proCDTbΔD4 was mixed with a 2-fold molar excess of BINTOXB/9 IgG or BINTOXB/22 LCmod IgG, respectively. Mixtures were allowed to bind for 20 min at room temperature. Complexes were then bound to protein A agarose (Thermo Fisher Scientific) and washed. Fab-antigen complexes were then eluted by rotating the resin in buffer containing 1:20 (wt/wt) HRV 3C protease relative to IgG. The complex of D4 with BINTOXB/9 was further purified via passage over a Superdex 200 Increase 10/300 GL (Cytiva) SEC column in 2 mM Tris (pH 8), 200 mM NaCl, 0.02% NaN_3_. The complex of proCDTbΔD4 with BINTOXB/22 was further purified via passage over a Superdex 200 Increase 10/300 GL (Cytiva) SEC column in 20 mM Tris (pH 8), 150 mM NaCl, 6 mM CaCl_2_. The same procedure was used for the immunoprecipitation of proCDTb using BINTOXB/19 (Fig. S1B), except that SEC was performed using a HiLoad 16/600 Superdex 200-pg column (Cytiva).

### CDT neutralization assay.

Human colonic epithelial cells (HT29 cell line) cultured at 37°C with 5% CO2 in Dulbecco’s modified Eagle’s medium (DMEM), 10% fetal bovine serum, 1% glutamine, 1% antibiotics (penicillin-streptomycin-amphotericin) were seeded in 96-well black tissue culture plates (Greiner Bio-One) at a density of 4.103 cells/well. After 24 h, the medium was removed and 50-μL volumes of serial 3-fold dilutions (performed in the same medium as previously) of each monoclonal antibody, starting at a concentration of 303 μg/mL, were added, followed by the addition of 50 μL of a mix of CDTa (25 ng/mL) and cleaved, monomeric CDTb (75 ng/mL). Plates were then incubated at 37°C with 5% CO2 for 6 days. After removal of the medium, 100 μL of Hoechst dye (BD Pharmingen) diluted 1:500 in PBS was added to each well and allowed to incubate for 2 h at room temperature in the dark. The Hoechst dye was then removed from the wells and the surface covered by the fluorescent Hoechst staining was determined using the Scanlab image acquisition system and AxioVision software (Zeiss). The neutralizing activity of each monoclonal antibody was expressed as the antibody concentration inhibiting 50% of the cytotoxicity (EC_50_).

### Biolayer interferometry.

Purified proCDTb, proCDTbΔD4, and D4 at 200 nM were immobilized to Ni-NTA biosensors using an Octet RED96e (ForteBio). Loaded Ni-NTA biosensors were then dipped into 200 nM BINTOXB/22 Fab for 600 s to measure association, and then into buffer (20 mM Tris [pH 7.5], 150 mM NaCl, 6 mM CaCl_2_, 1 mg/mL BSA, 0.05% Tween 20) for 600 s to measure dissociation.

### Surface plasmon resonance.

To measure proCDTb Fab-binding kinetics (for BINTOXB/9 and BINTOXB/22) or proCDTb Fab-binding affinity (for BINTOXB/19), proCDTb containing a C-terminal 6×His tag was immobilized onto an NTA sensor chip (Cytiva) in a Biacore X100 (Cytiva) using a running buffer of 10 mM HEPES (pH 8.0), 150 mM NaCl, 6 mM CaCl_2_, and 0.05% Tween 20. Following immobilization of ~200 RU proCDTb, Fab solution (0.1, 1, 2, 5, 10, and 25 nM for BINTOXB/9 and BINTOXB/22; 39 nM, 78 nM, 156 nM, 313 nM, 625 nM, 1.25 μM, 2.5 μM, 5 μM, and 10 μM for BINTOXB/19) was flowed in and association was measured for 180 s. After association, running buffer was flowed in for 600 s to measure dissociation. Regeneration and re-functionalization of the NTA sensor chip was performed after each association and dissociation cycle using 0.35 M EDTA followed by 0.5 mM NiCl_2_. For BINTOXB/9 and BINTOXB/22, global association and dissociation rate constants were obtained by fitting the data to a 1:1 binding model. For BINTOXB/19, the equilibrium dissociation constant was obtained via hyperbolic fit of the concentration-dependent equilibrium response.

### X-ray crystallography.

The complex of D4+BINTOXB/9 Fab (0.1 μL) at 10.2 mg/mL in 2 mM Tris (pH 8), 200 mM NaCl, 0.02% NaN_3_ was mixed with 0.1 μL mother liquor (10.1% PEG-3350, 13.4% 2-propanol, 0.2 M ammonium citrate [pH 7.5]) using an NT8 (Formulatrix) and spotted onto a sitting-drop location in a well containing 60 μL mother liquor. A 2.6-Å resolution X-ray diffraction data set was obtained for D4+BINTOXB/9 using a crystal that was rapidly soaked in 10.1% PEG-3350, 13.4% 2-propanol, 0.2 M ammonium citrate (pH 7.5), and 20% ethylene glycol before being flash-frozen in liquid nitrogen. The complex of proCDTbΔD4+BINTOXB/22 LCmod Fab (0.1 μL) at 7.5 mg/mL in 20 mM Tris (pH 8), 150 mM NaCl, 6 mM CaCl_2_ was mixed with 0.1 μL mother liquor (30% PEG-8000, 0.09 M sodium nitrate, 0.09 M sodium phosphate dibasic, 0.09 M ammonium sulfate, 0.1 M imidazole [pH 6.5], 0.1 M MES [pH 6.5]) using an NT8 (Formulatrix) and spotted onto a sitting-drop location in a well containing 60 μL mother liquor. A 2.5-Å resolution X-ray diffraction data set was obtained for proCDTbΔD4+BINTOXB/22 LCmod Fab using a crystal that was directly flash-frozen in liquid nitrogen. Diffraction data were collected at the SBC-19ID beamline (Advanced Photon Source, Argonne National Laboratory). Indexing and integration of the diffraction data were performed in iMOSFLM (39), which were then scaled and merged using AIMLESS (40). Molecular replacement was performed with Phaser (41), with subsequent iterative rounds of model building and refinement in Coot (42), Phenix (43), and ISOLDE (44). Data collection and refinement statistics can be found in Table S1. Structural biology applications used in this project were compiled and configured by SBGrid (45).

### CDTb oligomerization assay.

For each reaction, 500 μg of proCDTb was mixed with Fab (BINTOXB/9, BINTOXN/19, or BINTOXB/22; 2-fold molar excess relative to proCTDb monomers) or buffer (for trypsinization in the absence of Fab) at a final concentration of 1 mg/mL proCDTb in 20 mM Tris (pH 8), 100 mM NaCl, 6 mM CaCl_2_. Trypsin from bovine pancreas (Sigma-Aldrich) was added at 1:20 (wt/wt) relative to proCDTb (25 μg) and the reaction mixture was incubated at 37°C for 20 min. The reaction was then run over a Superdex 200 Increase 10/300 GL (Cytiva) SEC column in 20 mM Tris (pH 8), 100 mM NaCl, 6 mM CaCl_2_.

### Negative-stain electron microscopy.

BINTOXB/9+proCDTb at 0.03 mg/mL or CDTb double heptamer (from reaction with no Fab or BINTOXB/19 Fab) at 0.05 mg/mL was applied to CF400-Cu grids (Electron Microscopy Sciences) that were plasma-cleaned for 30 s in a Gatan Solarus 950 with a 4:1 O_2_:H_2_ ratio and allowed to bind to the support for 30 s. The grid was then washed twice with 20 mM Tris (pH 8), 150 mM NaCl, 6 mM CaCl_2_ and then stained with NanoW (Nanoprobes). Grids were imaged in an FEI Talos transmission electron microscope (Thermo Fisher Scientific) using a Ceta16M detector. Micrographs were collected at a magnification of ×92,000, corresponding to a pixel size of 1.63 Å/pixel. Contrast transfer function estimation, particle picking, 2D classification, *ab initio* reconstruction, heterogeneous refinement, and homogeneous refinement were performed in CryoSPARC v3.3.1 (46).
