# Supplementary material for: Structural Basis for Binding of Neutralizing Antibodies to Clostridioides difficile Binary Toxin
Source: J Bacteriol. 2023 Mar 23;205(4):e00456-22. doi: 10.1128/jb.00456-22 (PMC10127577; doi:10.1128/jb.00456-22)
Supplement: Supplemental file 1 — Fig. S1 to S5 and Table S1. Download jb.00456-22-s0001.pdf, PDF file, 0.8 MB [file jb.00456-22-s0001.pdf]

Figure S1

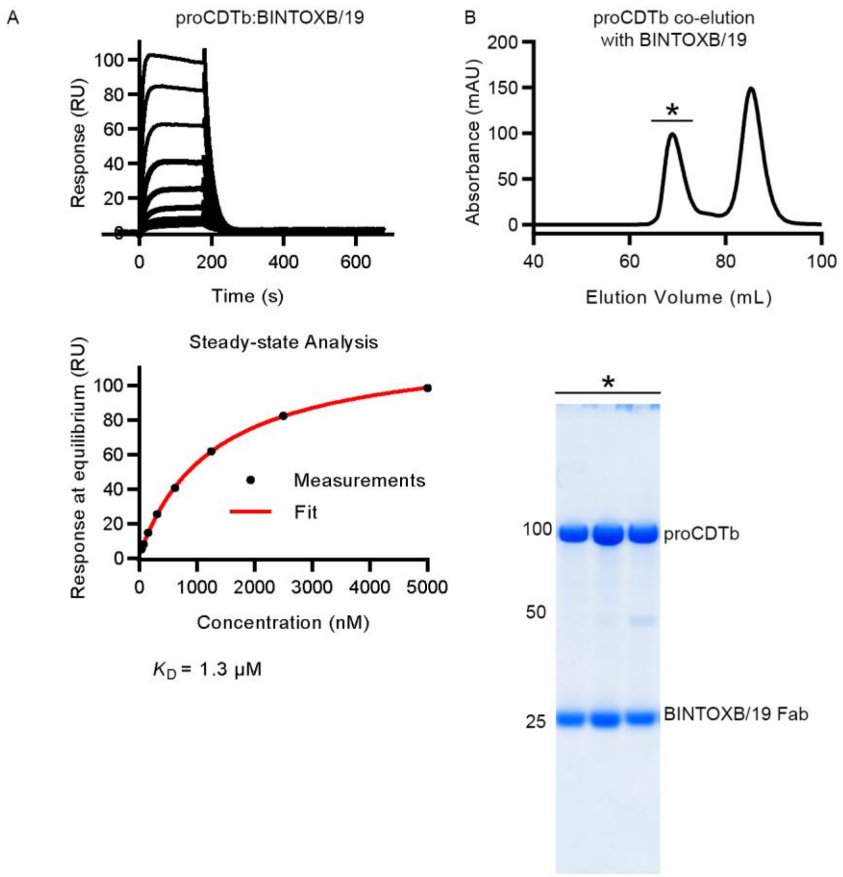

Figure S1. Binding of BINTOXB/19 to proCDTb.

(A) The top plot shows the surface plasmon resonance sensorgrams for BINTOXB/19 Fab binding to proCDTb. The association phase is 180s and the dissociation phase is 600s. The bottom plot shows the steady-state analysis of the BINTOXB/19-CDTb binding equilibrium. Points show the equilibrium response for each concentration of BINTOXB/19. The red line shows the hyperbolic fit used to determine the dissociation constant. (B) SEC profile of proCDTb in the presence of excess BINTOXB/19 Fab obtained by immunoprecipitation with BINTOXB/19 IgG and subsequent Fab cleavage. The peak containing the proCDTb+BINTOXB/19 Fab complex is labeled with a star. Below the SEC profile, an SDS-PAGE gel is shown for the proCDTb+BINTOXB/19 Fab complex peak. Molecular weights in kilodaltons are labeled on the left and band identities are labeled on the right.

Figure S2

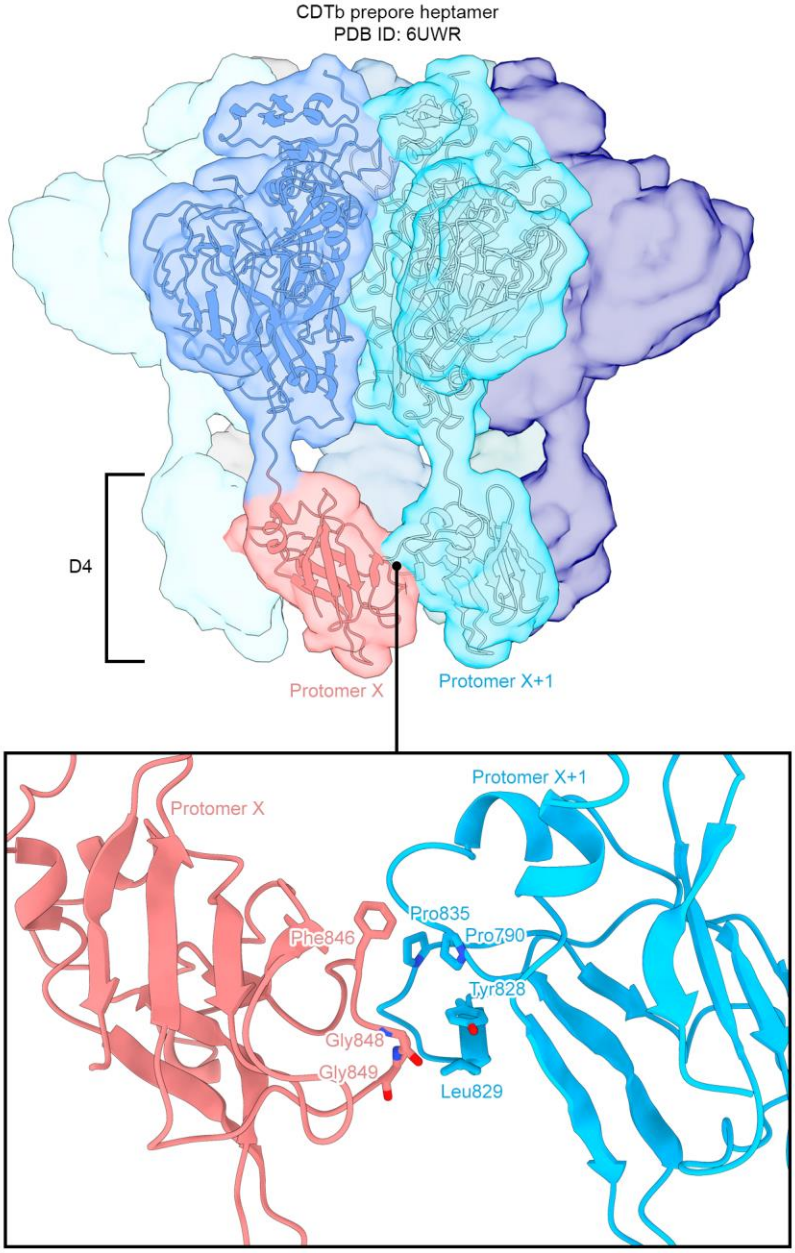

Figure S2. D4-D4 interface in the mature CDTb heptamer.

On top, a model of the cleaved CDTb prepore heptamer (PDB ID: 6UWR) is displayed with each protomer shown as a molecular surface. Protomer X and protomer X+1 are shown as both ribbons and molecular surfaces. For protomer X, D1-D3′ colored blue and D4 colored pink. Protomer X+1 is colored light blue. A black circle in the D4-D4 interface indicates the location of the zoomed-in view shown below. In the zoomed-in interface, protomer X and protomer X+1 are shown as ribbons. Key residues are shown as sticks, with oxygens colored red and nitrogens colored blue.

Figure S3

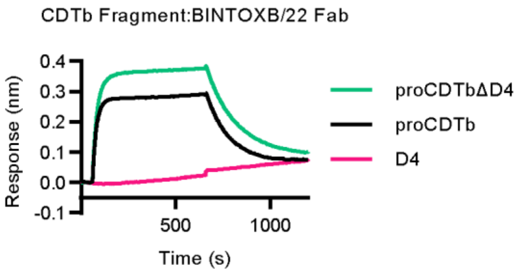

Figure S3. Binding of BINTOXB/22 to proCDTb fragments.
Biolayer interferometry measurements of proCTDb, proCDTbΔD4, or D4 captured on an NiNTA biosensor dipped into BINTOXB/22 Fab for 600s (association) and buffer for 600s (dissociation). Fab binding to proCDTb is shown in black, to proCDTbΔD4 shown in green, and to D4 shown in magenta.

Figure S4

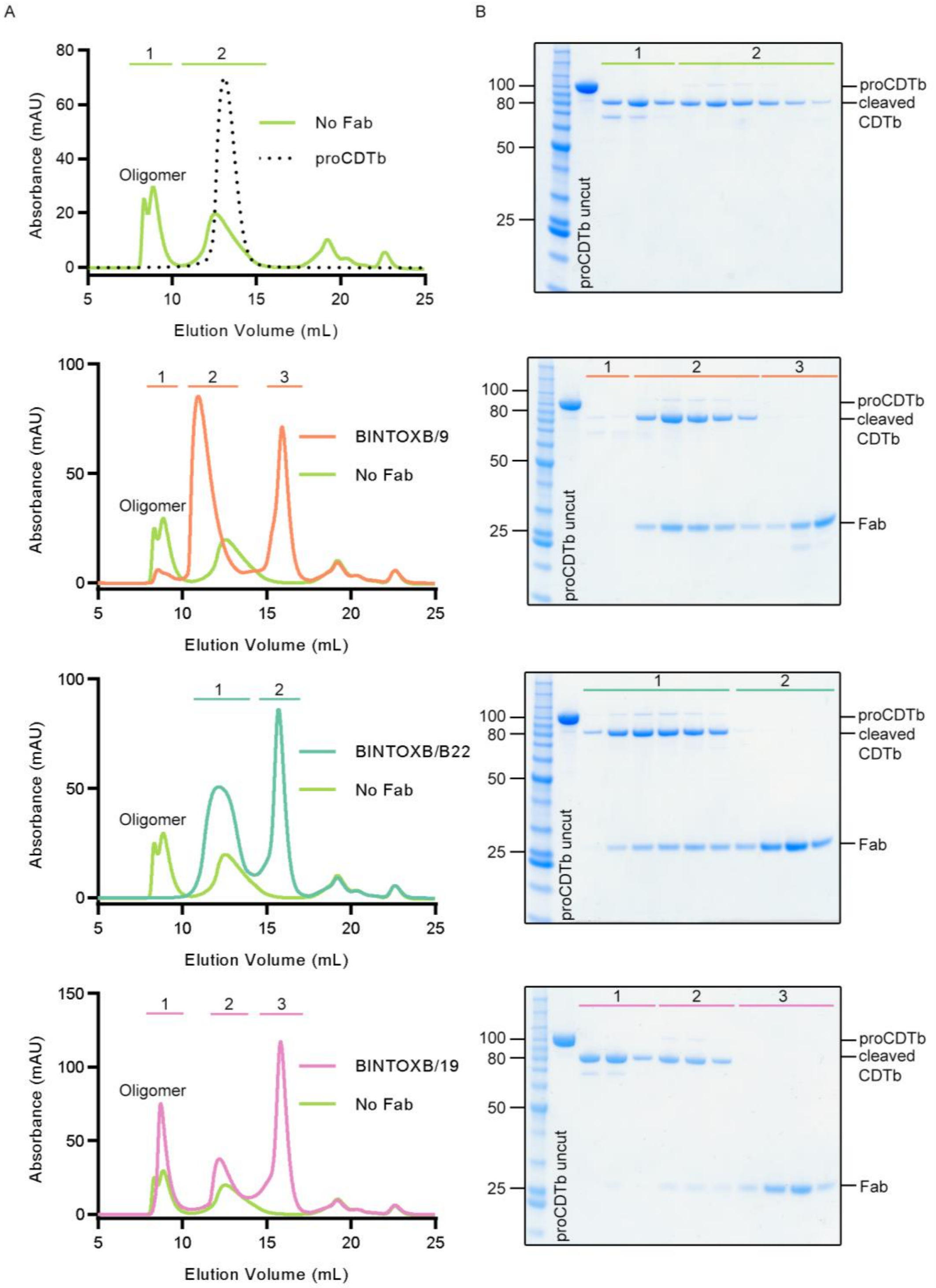

Figure S4. SEC and SDS-PAGE analysis of *in vitro* CDTb oligomerization.

(A) Full SEC profiles of proCDTb trypsinized in the absence or presence of Fabs. The trace for proCDTb trypsinized with no Fab is shown in green, overlaid with the trace for the non-trypsinized proCDTb shown as a dotted line. The trace for proCDTb trypsinized with BINTOXB/19 Fab is shown in pink, with BINTOXB/9 Fab in orange, and with BINTOXB/22 in teal. Each trace shown for reactions containing Fab is overlaid with the profile for the reaction containing no Fab (green). Numbered horizonal lines above peaks denote the samples in corresponding wells of the SDS-PAGE gels in (B). (B) SDS-PAGE gels of SEC fractions from trypsinization reactions shown in (A). Each gel contains molecular weight standards in lane 1, uncut proCDTb as a control in lane 2, and samples from the SEC peaks shown in (A). Molecular weights in kilodaltons are labeled on the left and band identities are labeled on the right.

Figure S5

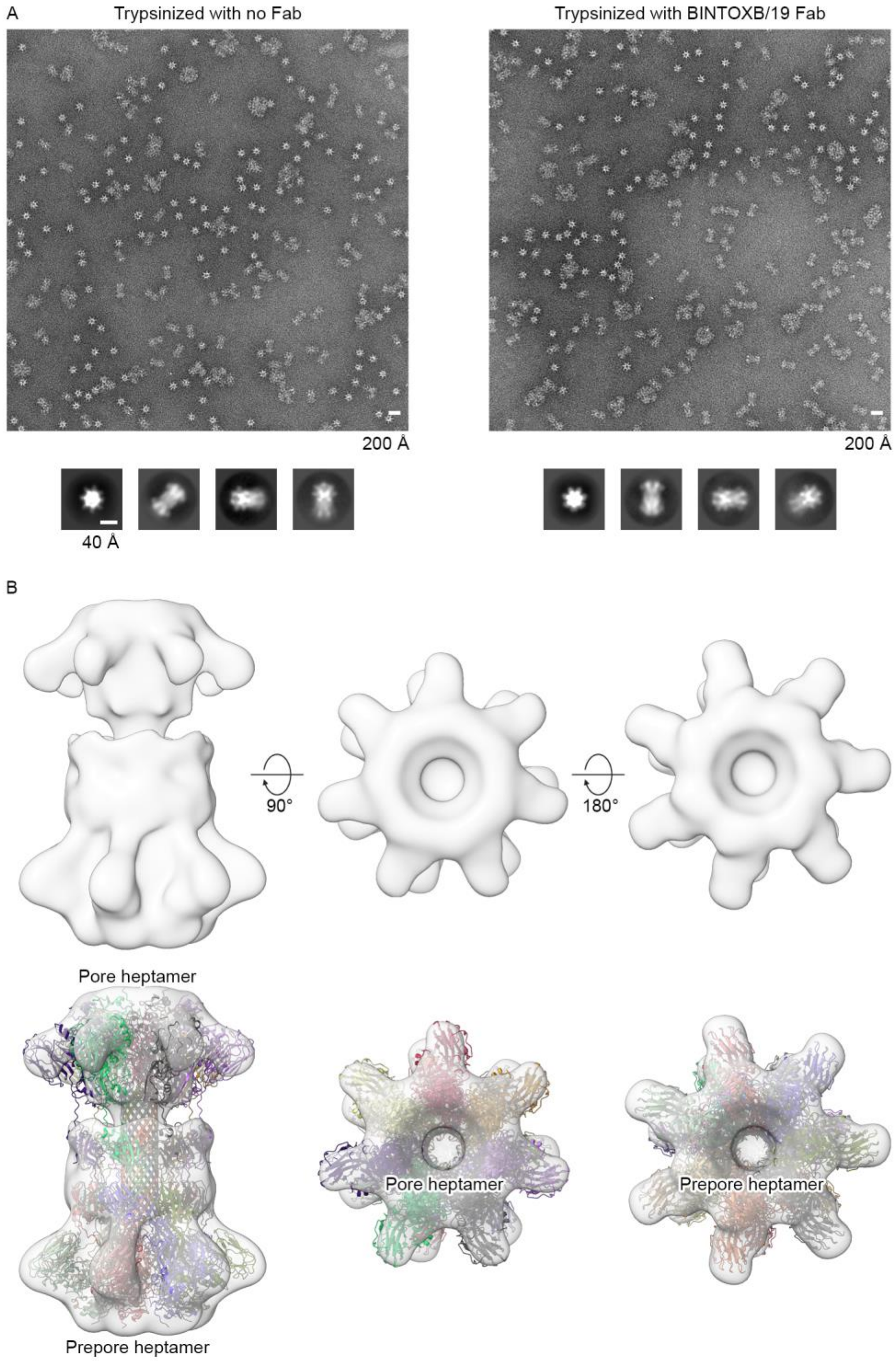

Figure S5. Negative-stain EM of CDTb double heptamers produced by trypsinization
(A) Negative-stain EM micrographs and 2D class averages for the CDTb double heptamer obtained after trypsinization in the absence of Fab (left) or in the presence of BINTOXB/19 Fab (right). (B) 3D reconstruction of the asymmetric CDTb double heptamer obtained by trypsinization in the absence of Fab. The map is shown without (top) and with (bottom) a fitted model of the CDTb double heptamer (PDB ID: 6UWR)(19). The prepore heptamer and the pore heptamer components of the double heptamer are labeled

Table 1. Crystallographic data collection and refinement statistics.

	BINTOXB/9+ CDTb D4	BINTOXB/22+ proCDTb ΔD4
**PDB ID**	8DCN	8DCM
**Data collection**		
Space group	$P2_122_1$	$P2_12_12_1$
Wavelength (Å)	0.979	0.979
Cell dimensions		
a, b, c (Å)	98.2, 111.8, 148.4	70.2, 118.5, 166.1
α, β, γ (°)	90, 90, 90	90, 90, 90
Resolution (Å)	98.21-2.60 (2.69-2.60)	96.46-2.50(2.58-2.50)
R_{merge}	0.127(1.155)	0.109 (0.440)
$I / \sigma I$	8.2(1.9)	10.5(4.7)
$CC_{1/2}$	0.991 (0.608)	0.990 (0.900)
Completeness (%)	99.4 (99.5)	94.3 (83.8)
Redundancy	5.7 (6.0)	5.4 (5.3)
Total reflections	287,086 (27,565)	246,435 (10,454)
Unique reflections	50,581 (4,574)	45,779 (3,686)
**Refinement**		
Resolution (Å)	55.88-2.60 (2.65-2.60)	48.23-2.50 (2.55-2.50)
Unique reflections	50,515 (2,776)	45,722 (2,511)
R_{work} / R_{free} (%)	22.7/26.0	19.8/22.7
No. atoms	8,726	9,076
Protein	8,650	8,813
Water	76	259
Ca^{2+}	-	3
Na^{+}	-	1
B-factors (Å^2)		
Protein	64.7	36.5
Water	52.0	32.3
Ca^{2+}	-	41.6
Na^{+}	-	40.1
R.m.s. deviations		
Bond lengths (Å)	0.003	0.003
Bond angles (°)	0.58	0.64
Ramachandran (%)		
Favored	98.0	96.3
Allowed	2.0	3.7
Outliers	0.0	0.0

Each dataset was collected from one crystal. *Values in parentheses are for highest-resolution shell.
